# Supplementary material for: Overexpression of BoLSU1 and BoLSU2 Confers Tolerance to Sulfur Deficiency in Arabidopsis by Manipulating Glucosinolate Metabolism
Source: Int J Mol Sci. 2023 Aug 31;24(17):13520. doi: 10.3390/ijms241713520 (PMC10487721; doi:10.3390/ijms241713520)
Supplement: Supplementary file 1 [file ijms-24-13520-s001.zip › ijms-2552510-supplementary.pdf]

Table S1 Nucleotide sequences of the CDS and promoter of *BoLSU1* and *BoLSU2*

Name	Sequence
BoLSU1-CDS	ATGGTGGAGGCGGAAGAGATGGAGGAGTTACGGAGGAGGAGC<br>AGAGAGCTCGAGAGAGAAGTAGAGGAGATGAAGACGGCTATG<br>TTGGAGTTGTGGCGGCGGACAGTGGTGGCAGAAGAGGCCGAG<br>GAGAGACTCTGCTCGCAGCTAGCGGAGCTGGAGGTCGAGTCTC<br>TAGATCAGGCTCGTGACTATCACGATCGTGTGGTCTTCCTCATGG<br>ATCAAATCTCACGTCTCTCTTCTTTGTCCGTCGTTTCCTAG
BoLSU2-CDS	ATGGGGAAAGGAGGAAATTTCGTGACGGTGGCGGCTTCTGAGG<br>TGGAGGAGCTACGACGGAAGAACGGAGAGATGGAAAAAGCGG<br>TGGAGGAAATGAGGAGAGAGATGTTGCAGCTGTGGCGACGGA<br>CGCAGGTGGCGGAAGAGGCTGAGGAGCGTCTCTGCTCTCAGCT<br>GGCCGAGCTCGAAGCCGAATCGCTCGACCAGGCGCGTGATTAC<br>CAATCTCGCATCCTCTTCCTCGTGAACGAACTCTCTCGTCTCTCC<br>TCATCCGATTTGGCCTCGCCCTAG
BoLSU1-Promoter	TCGGATCAAAGCCGCTAACAAGCAAAACTCGAATCTGTATCTCC<br>TAAGAACACTTTGCCGGAAATATGTACTGCAAAGATTGATATCG<br>TCGGAGGATAGAGATTGAGCATAACCAACAACTAGGAAGAAAC<br>TCCTAAGAAAGCAGCCGGAATATAACAAGAAGGAGAGAGCTGA<br>AGCTTTGATTAATCAACTAAACTCGGAGAGAAAAGGGACCAGA<br>GTTGCGTGGGTTCCCAAAGGACCCGAGTTCGAATCCGCACCAC<br>ACCAAATTTCCACGCGCGTGGCCACCGGGGCTTTCACATTCTTT<br>CTCCAGAGAATGGTTTACCATTTTTTTTAAACTCCGAGATACAA<br>GAAATGTCTTGAAGAAATTGTTCCGGTGTAGTTGTTATATATACA<br>CAACAAAACTCGCCTTGCGAAATTAAGATCCGATTTAGCCGAAA<br>GAGAATCCGCTAAAAGGGAAAGTGGAGTCAGACTAGCCGAAA<br>CACAAGATCAAAACAAAAAAACAAAAGAATTTTTATTCCTTCCT<br>CTTCGTCCCTGTGAAGGATATAGAGAGAGATAACAGGGAAGCG<br>AAGGGAATTGTGGTAGTAGCCCCAGTACTTTACTCGGTAATCAA<br>TGTATACCATTCATTTAAAAAAAAAATCAAAATTAAGGCTGAGA<br>GTGTTACACCACAGCCATATACTGCCATACATCTGATCACTGACG<br>CAAATACAAATCGACGGACCAAATTTACTGTGAGAAAATATTAT<br>GTTTATATAAGAAAGAACAAATATTATATCATTGAACTCCAGTAA<br>ACCACGGCACTGCGATTCTTGATTATTATATTAGGCCATCCCCAG<br>TGGCTCTTTTTTGTAAGAGTTTCTTGTGCTAAAAATAATACTCAA<br>AAGTATGAGAGAAACATTGGAAAAGTGGAGAAACGATTCAGAG<br>AGAAGAAACGTTTGACCCTCTTGAAGAAACCTATTCTACACATG<br>TAACTCCATCAAATTTTAACATTTTTAGTTTTAATTATAAAATTGT<br>TTAATGTTAAAGTATTATTAAACTTATTGTTGAGAGACTCAATTTA<br>AGAAACCATTACTAATGATGCTCTTACACCCTAATCGTATTGATA<br>CTGTACTATATATATCATATGAATAAAATTTTGTTTTATAAATAAGT<br>AAAAAACTGGAAATTCGGCTTTTTATTTTGTATGCATGATAAATT<br>AATGAATTTCAAATGTGATGGAAAAAAAATTGTTCATTTTGAAA<br>AAAAGCTTATATTTTATTTTCTTCTATTCTTTTGAAAATACAAGTT

	ATTTATTGTTCTCCGTAATAGTAGTATATAAGTCAGGAGTGGTTTA<br>AATTTCTAAGCAATTCTAAAGGAGAAAATGTTGACCCAAACAG<br>AAGATAGGAAAAAATACTCCCTCCGTTTCATTTTACTTTCGACA<br>CACAGATTAATAAAACATTTAAGTTTATCTATTTACTAGATAAAA<br>ATATCATTACCAATACACCTAACCAGATTTCAACCAATAGAAAAA<br>TAGATTAGAATAAAAAGTCAATAAATTTTGCATTGAAATCATAAA<br>ACGACACTAATTTTGAAACGAAAATTTTGCTCTAAAACGATATC<br>TAATTCGAAACGGAGGGAGTAAATATCAACAACAGATAGATTCA<br>TTGAACCTGGACAAGCTAAAGCTACCACACAAGATGCTTATTAA<br>AGGACATCGTCTAACCCTAACACTCTAACTTCAAAAAAGAAGA<br>AAACCAAACCACAATAATAAATCCTTTGTACGTAGAGACTGCAA<br>CTTATTTAATTAATTGCGAAAGGAGGTTACGTGA
BoLSU2-Promoter	GGGAGTAAATAGTTGAATAATCGTGACGGCCATTGACTTTGATA<br>GAGTCTGTCCAATTAAGAGAAGGAGGTGGGAATATGATTACGTC<br>GACAAGGCTTTGCTCTCATAACCATTCCTTTACGGTGGCTTAAAT<br>TGGCGGAATATATTAGTGGAAAGGATAAAGGTTAGAAAATGATT<br>TCCACTACATTCAATTTTTTTTCCTAGATACAAGAAAAGTAGCGA<br>AATTTTTGTTTTCTTTTCTCCTTATTACATGTTTTTTTTTTTTTTTTT<br>TTTTTTTTTTGCTAAATGGTAAATATCATTAAGAAGATAATGAAG<br>ATCCGGTTACAAGTTAATGAAACCAAGAGATGCTTTTCAGCCAA<br>TCAGGTTTAAGGGGTTGCAAAAAGTTAAAAAAAACCCCAAAGA<br>ACCTACAAAATTAACATCATGCAACCCAGACAATGGTTTACGCG<br>AAGTTAATACAACATTGCAGCACAAAGACAACATTTTGGGTTCT<br>CATACCAAAACGCTTCTTGTTCATCTGTAACAAAACAGAGAACA<br>AGAGAATAACCTGAAGGCAAGATTGATCGAGCCTCACCGAAGT<br>GGCTCCTAGTAAGCATCGACGGAAGCGCCTGGTTCACGCCCCG<br>GAGCTCCGCAGGAGGAGGACGTCAACAAAGCAAGCCCAAGCT<br>AACGCAGAAGTTCGCTAGAATGTAAACCTGCATGCATACTACAC<br>CGCTGAAGATGAAGAACGCGGTCAAGTGCAGTCAGTCGTTATG<br>GCCTCATTCCGCTGCGCGACACAGAAGATGCAGCGAAGACAGC<br>AAGGTTACAAAACATCAATCGACCGGGAGCTTTGGAATGGGAG<br>ATGAAGCACGGAAGGAGCACAGAACTCCTTGAAACAGGTGAC<br>CTGAAGGCGAACGAAAGCATCGCCCTATCCGCAAGAGACCTCG<br>ACGTCGAGGGTAGTCCTCAAGACCCTTTTGAAGACGATGAGAG<br>CGGGGAAGAGGAGACAAACCTTCGCTGAGGAACACCATGGAA<br>GCCGTCTTTTGACCTGTTTTTTTTTTTTTTTTGAGGAAGGGCATTC<br>TTTATTACATGTTACAAACAAACATGTCATCTAAGAGACATTCCA<br>AAAATATAGAAAGAAATTCGTATAACATCTCCCTTGCAAAGAAA<br>AGTATCTGGTCCAAACTTTTTTAGATTTACTAATGTGTATTCTAGG<br>AACACGGCTTCTGACAGAGAGTATTTCCACATATCATGGGCCTA<br>TAGCGAGTTTACGCCGTAGACTAATATCGTTTGTGTATATGCAGA<br>ATTCTTTTTGTAGAATTTTCAAAATAGTATCATAAAATTTTGAATT<br>TCTTTTAGCAAAAATAATAATTGTATATCCAGAAATTTCCCGTGA<br>AACTTTTTTAATTCTCCAACAACATTTCTAGCCGCTGAAATAATT

	TTGTCGTACCACTAAAATTTTGGAAAAATATATGGTGGGATCCAA CGTGTGTCAGAAAACAATAAAATAACAAATTGTTCTAGAGGAG GAACATAAAAAACGAGAACAAGAGATACATTGAACCTAGACAT ATACCCACCCAAAAGCCCAGTCACCATGATGCCTATAAAATAGG AGCATTCAAGAACAAGA

Table S2 Sequences of primers used in this study

Name	Sequence
ProBoLSU1-F	TCGGATCAAAGCCGCTAA
ProBoLSU1-R	CACGTAACCTCCTTTCGCA
ProBoLSU2-F	GGGAGTAAATAGTTGAATAATCGTG
ProBoLSU2-R	TCTTGTTCTTGAATGCTCCTAT
BoLSU1-F	ATGGTGGAGGCGGAAGAGAT
BoLSU1-R	CTAGGAAACGACGGACAAAGAA
BoLSU2-F	ATGGGGAAAGGAGGAAATTTC
BoLSU2-R	CTAGGGCGAGGCCAAATCGG
BoACTIN2-qPCR-F	CAGTTACAATCGTACCTTC
BoACTIN2- qPCR-R	GATCATGGAATCCACGT
ACTIN2- qPCR-F	TTACCCGATGGGCAAGTC
ACTIN2- qPCR-R	GCTCATACGGTCAGCGATAC
MYB28- qPCR-F	TCCCTGACAAATACTCTTGCTGAAT
MYB28- qPCR-R	CATTGTGGTTATCTCCTCCGAATT
MYB29- qPCR-F	CAATACTGGAGGAGGATATAACC
MYB29- qPCR-R	AGTTCTTGTCGTCATAATCTTGG
MYB76- qPCR-F	ACGTTTAATCGATGATGGCA
MYB76- qPCR-R	ATGGGCTCAACTGGATTAGG
BCAT4- qPCR-F	CAGAAGATGGTCGGATTCTGCTA
BCAT4- qPCR-R	GGCAAAAGCTGTGAAGGTGGT
MAM1- qPCR-F	AATTCGGAGAACTCGTGGCCT
MAM1- qPCR-R	GCTCCCGCACATATACCGGAT
CYP79F1- qPCR-F	CCATACCCTTTTCACATCCTACTAGTCT
CYP79F1- qPCR-R	GTAGATTGCCGAGGATGGGC
CYP79F2- qPCR-F	CCCATAATAGACGAGAGGGTCGAAA
CYP79F2- qPCR-R	CGATCGCTGCTATACAAAATTCG
CYP83A1- qPCR-F	TTCAAGAGGTTGTCAATGAGACGC
CYP83A1- qPCR-R	CTACAATATCCAAGATGACGGCTTT
FMO_{GSOX1}- qPCR-F	CTTCTACTCTCCTCAGTGGCAAA
FMO_{GSOX1}- qPCR-R	CTAATGTCGT-CCCATCTTCAAAC
BGLU28- qPCR-F	TTGCCACTGAACTAGATTGGCA
BGLU28- qPCR-R	GGATCGGCTTGTGGAATATGAG
BGLU30- qPCR-F	GAATTAATGACAACGACGACGG
BGLU30- qPCR-R	TTACGTCACACCCATCTTCCAC
TGG1- qPCR-F	GACTTTGAGAAGGCTACTGCCGATTA
TGG1- qPCR-R	CCATTTGCCAGATGCTTTGAGGT

MYB51- qPCR-F	TCAACGAGTTCTTCCTTCGCA
MYB51- qPCR-R	ACGGAGGAATCAGAGAACGTG
MYB34- qPCR-F	CACGACTGTCGATAATTTTGGGTTT
MYB34- qPCR-R	CATATTGTCATCTTCGTTCCAGGAA
CYP79B2- qPCR-F	AACAAAAAGAAACCGTATCTGCCAC
CYP79B2- qPCR-R	TCCTAACTTCACGCATGCTATCTC
CYP79B3- qPCR-F	CTCCTTCTTCCTTGCAAATGGA
CYP79B3- qPCR-R	GAGAATCATCAAGAAGCAAAGGG
PEN2- qPCR-F	CTGAAAACGGGTATGGTGAAGTAGC
PEN2- qPCR-R	TCCACTCGAAGTTATCTAGCAATGACC
PYK10- qPCR-F	CGCATTTCCGGTAAGCTTC
PYK10- qPCR -R	AAAGGCACCTGGTCGTTGCT
